# Advertising for Probationers

**Published:** 1920-10-09

**Authors:** 


### Advertising for Probationers.

We publish this week a suggestion made by a matron of wide experience in favour of a central system for recruiting probationers. Her proposal is that the State, presumably the Ministry of Health, should undertake this business for hospitals and infirmaries, and she outlines an interesting scheme whereby the candidates could be distributed to the schools with vacancies. There can be little doubt that, in spite of the natural preference shown by committees to secure probationers for themselves, some such central scheme is highly desirable. Always there will be local sources and influences at work tending to draw probationers into particular institutions. It is only for part of the staff that such schemes could operate. But every matron would, we think, value the privilege of sending in a list of her requirements to a central office could she be sure of this being followed in due course by well-sifted applicants.

We have never for our part ceased to look to the College of Nursing to inaugurate a central system of obtaining probationers. The State, including the Ministry of Health, is apt to look to voluntary agencies to act as taster in any new scheme. It has to be proved whether the right kind of woman can be brought to apply to a central office, and whether the hospitals will take to the theory of co-operation. To begin on a small scale and invite applicants to come forward would involve merely the setting aside of a room for the purpose and the engagement of a trained sister to interview them. This to begin with. But within a little while it is probable that the need would arise for testing candidates. Some form of preparation, such as classes in first-aid and bandaging, is found a most useful preliminary test for Red Cross workers. Why should it not be equally effective in filtering the heroines who desire to become trained nurses? The need is a very real one. If the College were to come forward and essay the task of recruiting, we believe it might open a new era in drawing women to become nurses.

